# Effects of Oxidized Phospholipids on Gene Expression in RAW 264.7 Macrophages: A Microarray Study

**DOI:** 10.1371/journal.pone.0110486

**Published:** 2014-10-21

**Authors:** Daniel Koller, Hubert Hackl, Juliane Gertrude Bogner-Strauß, Albin Hermetter

**Affiliations:** 1 Institute of Biochemistry, Graz University of Technology, Graz, Austria; 2 Biocenter, Division of Bioinformatics, Innsbruck Medical University, Innsbruck, Austria; University of Memphis, United States of America

## Abstract

Oxidized phospholipids (oxPLs) are components of oxidized LDL (oxLDL). It is known that oxLDL activates expression of a series of atherogenic genes and their oxPLs contribute to their biological activities. In this study we present the effects of 1-palmitoyl-2-glutaroyl-*sn*-glycero-3-phosphocholine (PGPC) and 1-palmitoyl-2-(5-oxovaleroyl)-*sn*-glycero-3-phosphocholine (POVPC) on gene expression in RAW 264.7 macrophages using cDNA microarrays. PGPC affected the regulation of 146 genes, whereas POVPC showed only very minor effects. PGPC preferentially influenced expression of genes related to cell death, angiogenesis, cholesterol efflux, procoagulant mechanisms, atherogenesis, inflammation, and cell cycle. Many of these effects are known from studies with oxLDL or oxidized 1-hexadecanoyl-2-eicosatetra-5′,8′,11′,14′-enoyl-sn-glycero-3-phosphocholine (oxPAPC), containing PGPC in addition to other oxPL species. It is known that POVPC efficiently reacts with proteins by Schiff base formation, whereas PGPC only physically interacts with its biological targets. POVPC seems to affect cell physiology to a great extent on the protein level, whereas PGPC gives rise to both the modulation of protein function and regulation on the transcriptional level.

## Introduction

OxPLs are components of oxLDL which plays an important role in atherogenesis. This lipoprotein particle interacts with the cells of the arterial wall leading to cell-specific pathophysiological consequences. The uptake of oxLDL by macrophages is a hallmark of atherosclerosis. Accumulation of the particle contents in these cells gives rise to the formation of foam cells and eventually leads to cell death by apoptosis and necrosis. It has already been established that the truncated oxidized phospholipids PGPC and POVPC are toxic components of oxLDL and induce apoptosis in cultured macrophages and vascular smooth muscle cells [1], [2], [3]. Both compounds are generated from PAPC under oxidative stress and are structurally very similar. They contain a long fatty acyl chain and a short carboxylic acid residue in positions *sn*-1 and −2, respectively. They only differ by the functional group at the -position of the *sn*-2 substituent which is an aldehyde moiety in POVPC and a carboxy group in PGPC [4]. The aldehydic function makes POVPC chemically reactive towards the amino groups of phospholipids and proteins and for that reason specifically affects the interaction of this oxPL with the cells. While PGPC is rapidly and efficiently internalized by cultured vascular cells, POVPC is retained in the plasma membrane for some time [5], [6], [7]. Therefore it has been speculated that POVPC might preferentially interact with the cellular components on the protein level, whereas PGPC could undergo a great variety of physical interactions with proteins and other biomolecules, e.g. by modulating gene expression directly or indirectly via transcription factors.

A series of studies has been performed to identify the effects of oxLDL and oxPAPC (a mixture of oxidized phospholipids) on the expression of selected genes. Hägg et al. found that oxLDL coordinately upregulated gene expression of the glutathione and thioredoxin system in human macrophages [8]. Hirose et al. detected different responses of human polarized macrophages to oxLDL depending on the activation state (M0, M1, M2) [9]. Almost 93% of the top 30 upregulated genes in M2 macrophages were also upregulated in M0 cells. However, all subsets of macrophages shared a certain number of upregulated genes. M0 and M2 cells showed high similarities of gene expression in top 10 functional ontology categories, whereas gene expression in M1 cells differed to some extent as compared to the other activation states. According to the study by Groeneweg et al., oxLDL increases the transcriptional response of murine macrophages to lipopolysaccharide-induced gene expression [10]. The genes induced by the oxidized phospholipid mixture oxPAPC in human aortic endothelial cells (HAECs) were identified by Reddy et al. [11]. Bochkov and Gargalovic et al. specifically studied the effects of oxPAPC on the genes of angiogenesis [12], [13]. Leitinger et al. identified the capacity of oxPAPC to induce expression of inflammatory genes in human vascular endothelial cells (HUVECs) and macrophages [14], [15].

Most of the studies listed above have in common that they explored subsets of the human or murine genome. In order to obtain more global information on oxPL effects on gene expression, we screened the murine transcriptome under the influence of PGPC and POVPC using microarrays displaying cDNA probes for most components of the entire mouse genome. In cultured RAW 264.7 macrophages, we found that PGPC upregulated a large number of genes relevant to atherosclerosis, whereas POVPC showed only minor effects on the transcriptional level. This result further supports the assumption that toxicity of oxPLs in vascular cells is dramatically altered by small functional differences in the oxidized acyl chain. The chemically reactive POVPC is efficiently scavenged by proteins and modifies their functions by direct contact whereas PGPC physically interacts with perhaps more molecular targets including those, modulating gene expression.

## Materials and Methods

### Materials

Oxidized phospholipids PGPC and POVPC were synthesized in our laboratory as described by A. Moumtzi [16]. Organic solvents and other chemicals were purchased from Carl Roth (Karlsruhe, Germany), Sigma-Aldrich (Steinheim, Germany) or Merck (Darmstadt, Germany). Tissue culture materials were purchased from Sarstedt (Nürmbrecht, Germany). Dulbecco’s modified Eagle medium (DMEM, 4,5 g/l Glucose) with phenol red and heat-inactivated fetal bovine serum were obtained from Invitrogen (Leek, Netherlands). PBS and cell culture supplements were purchased from PAA (Linz, Austria).

### Cell culture and RNA preparation

Macrophage-like cells RAW 264.7 (ATCC No. TIB-71, American Type Culture collection, Rockville, MD, USA) were routinely grown in DMEM (glucose 4,5 g/l, HEPES 25 mM, L-glutamine 4 mM, without sodium pyruvate) supplemented with 10% heat-inactivated fetal calf serum (FCShi) and penicillin/streptomycin 100 U/ml at 37°C in humidified atmosphere containing CO_2_ (5%). These cells have been used as M1 like cells throughout all experiments [17].

Cells were grown to 80% confluency in DMEM supplemented with 10% heat-inactivated FCS (v/v). Aqueous lipid dispersions were prepared using the ethanol injection method [18] in DMEM without heat-inactivated FCS. The final EtOH concentration of the incubation media did not exceed 1% (v/v). Cells were incubated with lipid dispersions for 1 h, 2 h, or 4 h, concomitantly with a reference culture, which was treated with DMEM containing 1% EtOH (v/v). Cells were harvested at 1, 2, or 4 h of incubation, washed with PBS and mRNA isolation was performed using the RNeasy Mini Kit from QIAGEN (Venlo, Netherlands) according to the procedure described for animal cells in the RNeasy Mini Handbook (Fourth Edition). The RNA of cells grown in a 58 cm^2^ culture dish was isolated using two columns and pooled afterwards. mRNA quantity and quality was checked using the Agilent 2100 Bioanalyzer RNA assay. Results are presented as means of three independent experiments (biological replicates) and are refered to control samples (incubation of cells with oxPL-free medium).

### Microarray analysis

The used mouse cDNA microarrays (>27 k elements), labelling and hybridization procedures were described previously (Pinent et al. 2005, Hackl et al. 2005). Briefly, cDNA was prepared from 25 µg total RNA with Random Hexamers and Superscript Reverse Transcriptase II, in the presence of amino allyl dUTP. cDNA samples were purified with QIAquick kit from QIAGEN, according to the manufactureŕs instructions, but using potassium phosphate wash and elution buffer instead of supplied buffers. N-Hydroxysuccinimide esters of Cy3 and Cy5 were coupled to the aadUTP incorporated in the cDNA. Coupling reactions were quenched by 0.1 M sodium acetate (pH = 5.2) and unincorporated dyes were removed using QIAquick columns (QIAGEN). Fluorescent samples were dried, resuspended in hybridization buffer (50% formamide, 5 * SSC, 0.1% SDS) and combined. In all Cot1 DNA and 20 µg poly(A) DNA were added and denaturated at 95°C for 3 min. The samples were applied to the prehybridized slide (incubation at 42°C for 45 min in 5 * SSC, 0.1% SDS) and hybridized in a humidified chamber overnight at 42°C in the dark. The slides were washed at room temperature twice for 2 min in a 1 * SSC, 0.2% SDS solution, for 4 min in 0.1 * SSC, 0.2% SDS, for 4 min in 0.1 * SSC and for further 2 min in 0.1 * SSC and dipped twice in MQ water. The slides were dried and scanned with a GenePix 4000B microarray scanner (Axon Instruments) and the resulting TIFF images were analysed with GenePix Pro 4.1 (Axon instruments). Microarray analyses were performed in triplicates with dye swap, corresponding to three independent experiments (biological replicates). Features were filtered for low-quality spots and arrays were global mean and dye-swap normalized, and log2-transformed using ArrayNorm [19]. Genes with >1.5 fold change and adjusted p-value (FDR) <0.05 were considered significantly differentially expressed using the R/Bioconductor package *limma*. Microarray data are available in the ArrayExpress database (www.ebi.ac.uk/arrayexpress) under accession number E-MTAB-2781.

### Bioinformatics analysis

**Figure 1:**
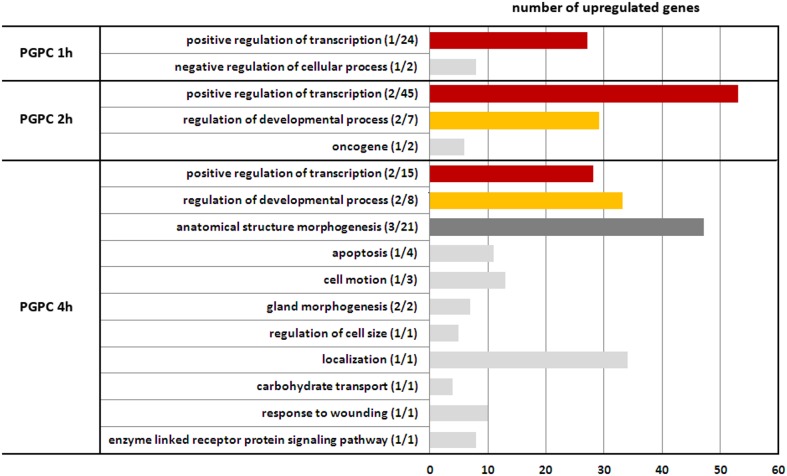
Time dependent effects of PGPC on gene expression in RAW 264.7 macrophages. An annotation term clustering with annotation terms referred to biological processes of upregulated genes was performed with DAVID. Dominant biological processes were classified applying analysis steps described in “Materials and Methods”.

Significantly up or down regulated genes were analysed using DAVID [20], [21], which systematically maps these genes to associated biological terms (i.e. gene ontology) and analyses if annotation terms are significantly enriched (over represented) compared to chance (background population of all known mouse genes from the Gene database). As POVPC showed only very minor effects on the gene expression levels, we analysed regulated genes of RAW 264.7 macrophages exposed to PGPC stress conditions at varying incubation times, which allows a time dependent view on the cellular responses. Furthermore we focused our analysis on biological responses and therefore restricted the analysis to annotation terms referred to biological processes using “GOTERM_BP_ALL” and “PANTHER_BP_ALL” as data sources. Clusters (groups) of enriched annotation terms sharing common genes were identified using medium classification stringency setting based on kappa statistics. As a result similar enriched annotation terms were grouped reflecting the same (or related) biological theme (e.g. “GO:0006915∼apoptosis”, “GO:0012501∼programmed cell death”). Annotation clusters were further pooled sharing very similar and significantly enriched annotation terms (FDR<20%) and interpreted with reflecting biological processes (see Figure 1). We selected 36 highly significantly regulated genes often listed in annotation clusters and considered as potential key elements of the obtained biological processes and performed a literature search. 19 of these 36 candidate genes were selected for RT-qPCR verification and further biochemical interpretation. Heatmaps of expression levels (log_2_ fold changes) of deregulated genes were visualized using Genesis [22].

### Quantitative RT-PCR

Microarray results of 19 genes were verified by quantitative real-time PCR. 1 µg of total RNA of biological replicates from microarray experiments was reverse transcribed into cDNA with GoScript Reverse Transcription System from Promega (Wisconsin, USA) according to the technical manual. cDNA was diluted to 1 ng/µl, 4.5 ng cDNA, primers and SYBR Green (Applied Biosystems; Carlsbad, USA) were transferred in a 96-well plate and RT-qPCR measurement was performed with ABI PRISM 7000 Sequence Detection System and hypoxanthin-guanin-phosphoribosyltransferase as the reference gene. Obtained results were analysed with QPCR webtool, developed at the Institute of Genomics and Bioinformatics [23]. Quantifications were performed in triplicates (technical replicates).

## Results and Discussion

**Figure 2:**
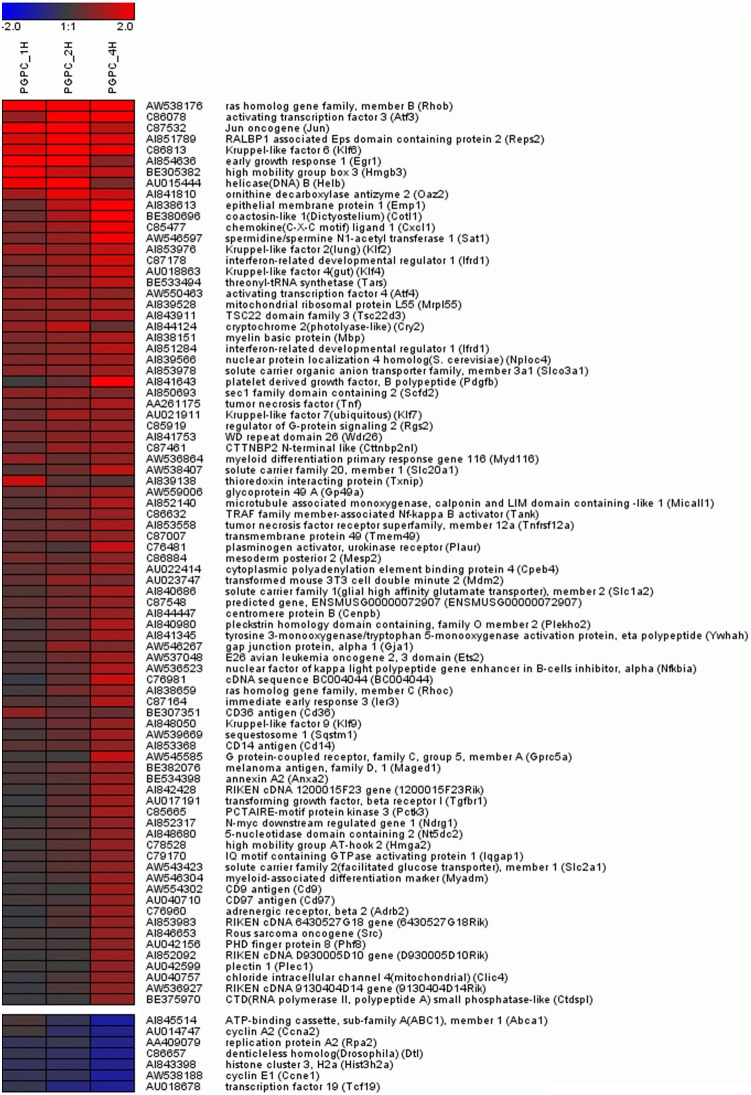
Time dependent heatmap of the effect of PGPC on gene expression in RAW 264.7 macrophages. Data are shown for unique genes with a FDR<5% and log_2_ fold change ≥0.8 or log_2_ fold change ≤ −0.8.

POVPC and PGPC are active compounds of oxLDL. The atherogenic effect of this particle is the sum of all the effects of all oxPL species. The aim of this study is to identify the contribution of two important chemically defined components of oxLDL, namely PGPC and POVPC. Here we report on a global microarray analysis of mouse genes that are upregulated or downregulated in response to the truncated oxidized phospholipids PGPC and POVPC. For this purpose, cells were incubated with 50 µM of oxPL followed by preparation of total RNA and expression analysis using mouse cDNA microarrays (>27 k elements) (ArrayExpress; E-MTAB-2781). From this data, we determined the total number of genes affected by either lipid. While PGPC affected 193 genes, POVPC only affected a small number of genes. The individual target genes were clustered and visualized as heatmap (Figure 2) displaying the time-dependent expression levels. A list of all genes affected by either oxPLs is provided in File S1. DAVID GO analysis was performed to identify biological functions of genes that were regulated under the influence of PGPC showing that the response of the transcriptome to this lipid largely matches the effects of its parent oxLDL (see Results and Discussion), which is a highly atherogenic particle. Results obtained from microarray analysis for genes that are related to atherogenesis were validated by qPCR.

**Figure 3:**
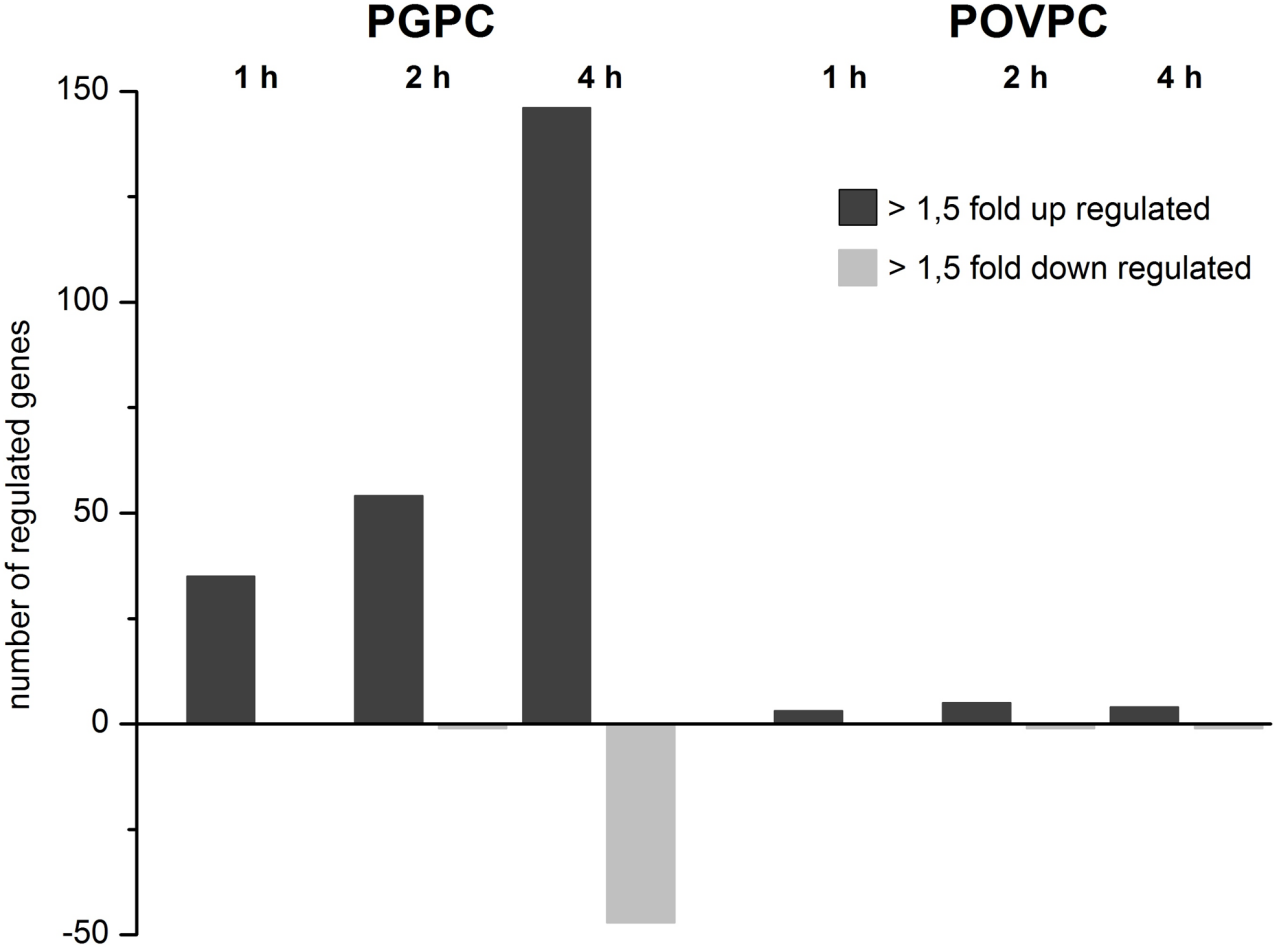
Time dependent effects of PGPC and POVPC on overall gene expression in RAW 264.7 macrophages. Number of significantly up- and down-regulated genes relative to control cells (FC>1.5; FDR<5%) are shown. PGPC: 146 up- and 47 down-regulated genes; POVPC: 4 up- and 1 down-regulated genes.

Figure 3 shows that PGPC influences the expression of a large number of genes. Within 4 hours, 146 genes are upregulated and 47 genes are downregulated under the influence of this lipid in a time-dependent manner. The number of expressed genes steadily increases in these cells upon incubation with this lipid for 4 hours. The individual genes upregulated by PGPC are listed in the heatmap shown in Figure 2. POVPC shows a much smaller effect on the transcriptional level. Only 4 genes are activated and one gene is downregulated if cells are exposed to this compound for 4 h. DAVID GO analysis of all genes that are upregulated by PGPC led to the identification of functional annotation term clusters. Many of these clusters contain elements that are relevant to the initiation and development of atherosclerosis that is developmental processes/angiogenesis, apoptosis, negative regulation of cellular processes/cell cycle, response to wounding/procoagulant and inflammatory mechanisms (see Figure 1). The individual biological functions of all genes affected by POVPC and selected genes influenced by PGPC were obtained from data bank search in Pubmed/Gene and are shown in Table 1 and Table 2, respectively. The PGPC-induced genes that are mainly involved in atherogenic processes and their molecular functions are shown in Table 3. Their physiological roles in mediating the toxic and atherogenic signal of this oxidized phospholipid are highlighted in the Discussion section.

**Table 1:** Time-dependent transcriptional effects of 50 µM POVPC in RAW 264.7 macrophages as observed with microarray analysis.

Gene symbol	RefSeq	POVPC 1 h	POVPC 2 h	POVPC 4 h	Description
Klf6*	NM_011803	↑	↑	-	Transcription factor
Hmgb3*	NM_008253	↑	-	-	HMG box (DNA binding) subfamily; DNA replication, nucleosome assembly and transcription
c- Jun*	NM_010591	↑	-	-	Member of AP-1 complex; Transcription regulator
Rgs2*	NM_009061	-	↑	-	Regulator of G protein signaling
Egr1*	NM_007913	-	↑	-	Transcriptional regulator; Differentiation and mitogenesis
Emp1*	NM_010128	-	↑	-	Epithelial membrane protein; cellular contact [62]
B4galt5	NM_019835	-	↓	-	β-1,4-galactosyltransferase
Atf3*	NM_007498	-	↑	↑	Activating transcription factor; cellular stress response
Pdgfb*	NM_011057	-	-	↑	Platelet derived growth factor
FAM20C	NM_030565	-	-	↑	Potential regulator of differentiation and function of hematopoietic tissue
Lpl	NM_008509	-	-	↑	Triglyceride hydrolase/bridging factor for receptor mediated lipoprotein uptake
ABCA1*	NM_013454	-	-	↓	Cholestol efflux pump

↑upregulated genes; ↓downregulated genes; -no effect; *These genes were also affected by PGPC.

**Table 2:** Comparison of relative gene expression levels (log_2_ fold changes) observed by microarray (µa) and RT-qPCR (qPCR) analyses.

Gene	RefSeq	µa	qPCR	Description
Atf3	NM_007498	2.95	2.65	cAMP-dependent transcription factor
Atf4	NM_009716	0.79	0.86	cAMP-dependent transcription factor
c-Jun	NM_010591	1.32	1.72	Member of the AP-1 protein complex; transcription factor; cellular stress response
Junb	NM_008416	0.70	0.95	Member of the AP-1 protein complex; transcription factor
Klf2	NM_008452	1.35	4.49	Zinc finger transcription factor; T-cell trafficking
Egr1	NM_007913	0.78	1.01	EGR family of C2H2-type zinc-finger proteins; transcriptional factor
Tnf-α	NM_013693	1.08	1.94	Proinflammatory cytokine
Trib3	NM_175093	0.59	1.49	Putative protein kinase; sensitization of cells to TNF- induced apoptosis; negative regulation of AKT1
Src	NM_009271	0.87	1.12	Tyrosine-protein kinase/proto-oncogene
Ldlr	NM_010700	0.71	0.50	Receptor-mediated endocytosis of LDL
Gadd45b	NM_008655	0.67	0.79	Activation of the p38/JNK pathway; Regulation of growth & apoptosis
Rhob	NM_007483	2.72	3.90	Ras homolog family member B; GTPase; cytokine trafficking and cell survival; Regulation of CD36 expression
Cd9	NM_007657	1.02	0.69	Transmembrane 4 superfamily; differentiation, adhesion, and signal transduction
Rtn4	NM_194052	0.70	0.54	ER associated reticulon family member
Abca1	NM_013454	−1.10	−1.31	ATP-binding cassette transporter; cholesterol efflux
Ccnb1	NM_172301	−0.55	−0.64	Regulatory protein involved in mitosis
Plk1	NM_011121	−0.62	−0.94	Serine/threonine protein kinase; Cell cycle control
Rad51	NM_011234	−0.67	−0.65	Cellular response to DNA damage
Cd36	NM_007643	0.82	0.02	Scavenger-receptor

Cells were incubated with PGPC for 4 hours and cDNA was prepared for microarray and RT-qPCR analysis as described in “Materials and Methods”. Cycle numbers for RT-qPCR were normalized to hypoxanthin-guanin-phosphoribosyltransferase as housekeeping gene. Gene expression levels of oxPL-treated samples are shown as folds of data for control cells. Up- and down-regulated genes are shown in red and green, respectively.

**Table 3:** Processes described to be affected by genes regulated in PGPC treated RAW264.7 macrophages.

Upregulated genes	Cell response	References
Tnf-α, Atf3, c-Jun, Jun-B	Apoptosis via TNF-α pathway	[26], [63], [27], [28], [29], [37]
c-Jun, Jun-B	Apoptosis via aSMase/Casp3	[24], [31]
Trib3	Apoptosis	[38], [39]
Atf4	Unfolded protein response and angiogenesis	[12], [64], [44]
Egr-1	Procoagulant mechanisms and plaque stability	[15], [52], [54]
CD9, RhoB, Src, c-Jun, Ldlr	OxLDL/LDL uptake and foam cell formation	[56], [58], [65], [66], [67]
**Downregulated genes**		
Abca1	Cholesterol efflux	[50], [51]
Ccn B1, Ccn F, Ccn A2, Cdc 5, Cdc A7, Ccd 6, Plk1	Proliferation and cell cycle	[59], [60], [61]

Expression of the atherogenic genes in response to PGPC was validated by qPCR. Instead of verifying the genes that were most highly regulated, we focused on genes that were defined as the most important targets for the individual categories (biological processes) in DAVID analysis. 18 out of 19 selected genes could be confirmed by qPCR. This score of verified genes is much better than the average values of ∼75% reported in the literature and supports our method of gene selection for qPCR.

So far only a limited number of studies is available that describe the transcriptional effects of PGPC, POVPC and the complex oxidized PAPC in cultured macrophages. However, the respective results are difficult to compare with ours, since different cell lines, incubation conditions, and procedures for data analysis have been used. In addition, microarrays were used for gene expression analysis that only contained limited sets of genes. Our study represents the first global analysis of gene expression in a murine macrophage-like cell line. Many genes that are regulated by the oxPLs are also affected by oxLDL in the same cell line. In addition, we found gene effects of oxPL that have not been reported for oxLDL so far. Since PGPC is a major oxidized phospholipid in this particle, we conclude that it is one of the components that mainly contribute to its atherogenic properties. PGPC contains only one long hydrophobic acyl chain and as a consequence easily exchanges between the surfaces of lipoproteins and cells. Therefore, its uptake by cells and its cellular toxicity is largely independent of the expression/activity of specific receptors.

In summary, we found fundamental differences between the effects of the oxPLs PGPC and POVPC on gene expression in cultured macrophages. POVPC only affected a relatively small number of genes, whereas PGPC influenced many more genes. This is surprising insofar as both oxPLs drive the cells towards the same endpoint, namely apoptosis although different pathways are utilized in this process [5]. However, the data from microarray analysis did not reveal any gene expression changes relevant to apoptosis induced by POVPC. This could be due to the fact that POVPC induced apoptotic signalling mainly operates on the proteome level (posttranslational modification, e.g. phosphorylation, proteolysis) finally leading to activation of caspase 3 [24]. We suppose that this difference in PGPC and POVPC activity is at least in part due to the fact that POVPC is chemically reactive and PGPC is not. POVPC contains an aldehyde function in position sn-2 which enables this compound to form Schiff bases with amino groups of proteins [6] and very likely also with amino groups of phospholipids [25]. Fluorescence microscopic observations are in line with this assumption. We found that an exogenously added fluorescent POVPC analogue was enriched in the plasma membrane, whereas a fluorescent PGPC derivative quickly entered the cell interior and mainly localized to subcellular membranes [7]. Thus, the primary signalling processes are very likely to be different and the availability for intracellular nuclear receptors will be too, provided there are any. Our data confirm that POVPC mainly acts on the proteome level [7], [6], whereas PGPC also affects the transcriptome. Specific proteins undergoing noncovalent interactions with the latter lipid have not been identified so far except some receptor proteins (e.g. CD36, PAF receptor, PPARγ).

It has to be noted that the gene expression data presented in this manuscript have been obtained with cells after incubation with pure oxPL in protein-free DMEM in order to maximize lipid stability. It is likely that the cellular response may be different if the oxPLs are presented in incubation medium containing BSA or FCS. POVPC forms covalent complexes with proteins [7] and the resulting new epitopes could elicit more robust responses by the macrophages.

**Figure 4:**
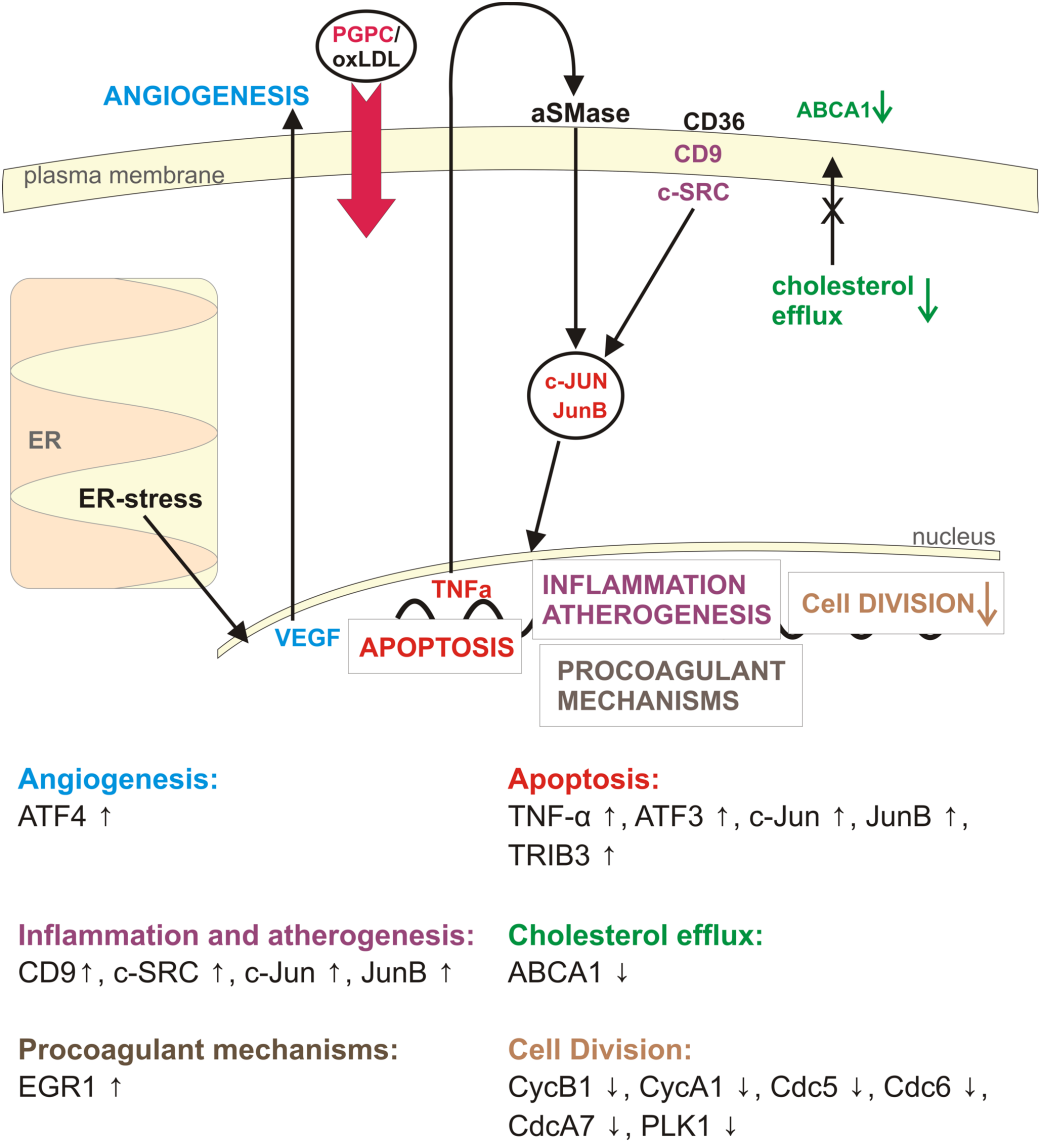
Biological processes and corresponding genes regulated by PGPC in RAW 264.7 macrophages.

The following sections mostly deal with the transcriptional effects of PGPC in macrophages encompassing hallmarks of atherogenesis including cell death, angiogenesis, cellular uptake and efflux of cholesterol, procoagulant mechanisms, plaque stability and last but not least cell cycle regulation (see Figure 4). Overall, the effects induced by PGPC and to a lesser extent by POVPC support the assumption that they are important biologically active components of atherogenic oxLDL which activates or inactivates the same genes that are affected by oxPLs.

### PGPC and cell death

PGPC treatment stimulated the expression of the cell death-associated genes c-Jun, JunB, TNF-α, ATF3, and Trib3 in mouse macrophages. These findings are in line with the observations that the same genes are activated by oxLDL in vascular cells pointing to a role of the oxPL as a toxic component of the modified lipoprotein. Jovinge et al. reported that exposure of monocytes/macrophages to oxLDL triggers expression and release of TNF-α [26]. This proinflammatory cytokine activates a series of genes in vascular cells involved in programmed cell death [27], [28]. The activity of TNF-α is intimately associated with the action/expression of the transcriptional factor ATF3 which is induced under stress conditions. Nawa et al. reported that this protein is highly expressed in endothelial cells and macrophages of human atherosclerotic lesions [29]. From *in vitro* experiments it can be inferred that it is involved in oxLDL-induced death of cultured HUVECs. In addition to ATF3, oxLDL stimulates the expression of TNF-α in these cells. If the ATF3 gene is silenced by antisense cDNA, TNF-α- and oxLDL induced apoptosis is substantially decreased. This data supports the assumption that oxLDL activates the TNF-α gene which after secretion stimulates ATF3 expression and cell death according to an autocrine mechanism [29]. This relationship is at least not fully applicable to PGPC toxicity. Nawa et al. measured gene expression after 24 hours incubation of the cells with oxLDL. Our microarray data were collected after much shorter incubation times. ATF3 expression already starts after one hour incubation with the oxPL, whereas activation of the TNF-α gene is observed after 2 and 4 hours. Obviously, different mechanisms may come into play under conditions of such short exposure times perhaps involving more direct interactions of PGPC.

A key element of apoptotic signaling is c-Jun. This protein is a component of the AP-1 complex representing a group of protein dimers that may be composed of members of the Jun family (c-Jun, JunB, JunD) and basic leucine zipper (bZIP) proteins [30]. Members of the Fos (c-Fos, FosB, Fra1, Fra2) and ATF families are the most important bZIP proteins for AP-1 complex formation. Expression of each AP-1 component is independently regulated. As a consequence, cells contain complex mixtures of AP-1 dimers with different functions depending on ambient conditions, e.g. oxidized phospholipid stress. AP-1 activity can be induced and modulated by a broad range of other extracellular stimuli including mitogens, hormones, extracellular matrix, and genotoxic agents [30]. Many of these stimuli including oxPLs and oxLDL [24], [31] also activate c-Jun N-terminal kinases (JNKs) which catalyze the phosphorylation and thereby increase the transcriptional activity of Jun proteins. Because AP-1 composition may vary to some extent, its activity is complex and may lead to controversial effects depending on experimental conditions. Nevertheless, increased levels of JUN and FOS proteins associated with high JNK activity have frequently been correlated with apoptosis [32], [33], [34]. Last but not least, there is a relationship with c-Jun activity and the oxLDL-induced expression of TNF-α and ATF3 [35], [36]. Takashiba et al. found a functional binding site for AP-1 at the TNF-α promoter, suggesting that c-Jun possibly regulates expression of this cytokine [37]. In addition, ATF3 seems to contribute to c-Jun activity, since both components synergistically activate death genes by binding to AP-1 and ATF-dependent promoters.

A particular aspect of oxLDL and oxPL-mediated apoptosis of vascular cells is the role of ceramide as a second lipid messenger. We found that the modified lipoprotein and the truncated oxidized phospholipids PGPC and POVPC induced apoptosis in cultured vascular smooth muscle cells and RAW macrophages. This phenomenon was associated with activation of aSMase within minutes, the formation of ceramide, the activation of JNK and p38 MAPkinase and the stimulation of caspase3. Since the respective kinase and caspase activities as well as apoptosis were reduced by a specific aSMase inhibitor, it was concluded that the latter enzyme was causally involved in lipid-induced cell death upstream of the other signaling components [24], [31]. In this context it is interesting to mention that TNF-α and ATF3 activities induced by oxLDL or PGPC also give rise to activation of JNK which phosphorylates apoptotic Jun proteins. However, it has to be emphasized that the aSMase pathway can be activated much faster. Exposure of cultured vascular cells to oxPLs or oxLDL activates the sphingolipid hydrolase within minutes, very likely on the protein level either due to direct lipid-protein interactions or modulation of enzyme activity by a general membrane effect.

More recently, TRIB3 was identified as a new protein which was expressed in macrophages in response to oxLDL in a dose-dependent manner [38]. This protein supposedly inhibits PKB activity and thus may be considered antiproliferative [39]. Since silencing of the Trib3 gene reduced the cellular susceptibility towards apoptosis, it was concluded that this protein is also causally related to lipoprotein-induced cell death. Our microarray analysis shows that PGPC also upregulates Trib3 expression and therefore further supports the hypothesis that PGPC is a component of oxLDL with apoptotic capacity.

### PGPC and angiogenesis

PGPC stimulated expression of the angiogenesis promoting transcription factor ATF4 in this microarray study. This result is in line with the observation that this gene is activated by a mixture of PAPC oxidation products in vascular cells pointing to a role of PGPC as a toxic component of oxidized LDL. The relationships are as follows:

DAVID GO analysis of our microarray data led to the identification of upregulated genes under the annotation terms “vasculature development“, “blood vessel morphogenesis“ and “blood vessel development”. Bochkov et al. reported that oxPL accumulation in atherosclerotic lesions stimulates plaque angiogenesis [12]. In addition, it has been shown that the density of *vasa vasorum* highly correlates with the amounts of mononuclear cells infiltrating the advanced lesions [40], [41], [42], [43]. On top of these observations, Bochkov et al. found that oxPAPC stimulates expression of VEGF in cultured HUVECs and monocyte-derived macrophages [12]. Since macrophages accumulate in atherosclerotic plaques that are rich in oxPLs, it may be speculated that VEGF secretion by these cells stimulates angiogenesis in a paracrine manner.

The same group found that expression of VEGF, and as a consequence angiogenesis, is induced by the unfolded protein response and the electrophilic stress response. Enhanced expression of VEGF by oxPAPC is accompanied by elevated levels of the transcription factor ATF4. In HUVECs, ATF4 is a key mediator of the unfolded protein response, promoting angiogenesis through VEGF activation in an autocrine manner [44]. ATF4 formation itself depends on activation of a stress-activated protein kinase. The eukaryotic translation initiating factor 2-α-kinase3 (PERK) selectively stimulates translation of ATF4, whereas it inhibits synthesis of most other proteins [43].

ATF4 may also be activated by the so-called electrophilic stress response which is induced e. g. by carbonyl groups in oxidized lipids and protects cells from such compounds [44]. Electrophilic stress response is initiated by oxidative modification of KEAP-1 which leads to release of the transcription factor NRF2 from the KEAP-1/NRF2 complex [45]. NRF2 binds to the promoter region of the antioxidant response element thereby stimulating expression of ATF4 which contains a putative antioxidant response element binding site. Silencing of NRF2 expression by siRNA abolishes oxPAPC-induced upregulation of ATF4 and VEGF [44]. In summary, these data provide evidence for a point of convergence of electrophilic and unfolded protein response pathways involved in the toxicity of oxidized phospholipids as pertinent to angiogenesis.

### OxPLs and cholesterol efflux

PGPC and POVPC treatment downregulated the expression of the Abca1 gene which mediates cellular cholesterol efflux. These results are at variance with the stimulatory effect of oxLDL on the expression of ABCA1 which is due to the presence of another subclass of OxPLs (9- and 13-HODE) and oxidized sterols [46]. Obviously, the lipid fraction of oxLDL contains a great variety of bioactive lipid oxidation products that may synergistically act or counteract in (vascular) cells.

Both ABCA1 and its counterpart ABCG1 mediate efflux of intracellular cholesterol, but sterol acceptors are different. ABCA1 promotes sterol transport to lipid-poor apoAI lipoproteins, whereas ABCG1 directs cholesterol to mature HDL and other lipoproteins [47], [48], [49]. The effects of cell and tissue specific inactivation of ABCA1 expression support the assumption that this gene inhibits foam cell formation and the development of atherosclerotic lesions [50]. According to experiments performed with LDLR^−/−^ mice, which received bone marrow from ABCA1 ko mice, ABCA1 deficiency can be compensated at least in part by ABCG1 [51]. Our microarray experiments show that downregulation of ABCA1 by oxPL is not compensated by ABCG1 at least on the expression level at the chosen time points.

### PGPC and procoagulant mechanisms

PGPC persistently upregulated expression of the proatherogenic transcription factor EGR-1, whereas POVPC only transiently activated this gene. The OxPL effect is in line with the observation that oxidized PAPC and oxLDL also increase expression of EGR-1 in HUVECs and RAW macrophages [15], [52].

EGR-1 (Early Growth Response-1) is a zinc-finger protein linked to maladaptive host response mechanisms and ischemic stress [53]. It is inflammatory and proatherogenic. In addition, its expression is stimulated during development of atherosclerosis in apoE-deficient mice. Further, EGR-1 knockout mice show smaller atherosclerotic lesions. EGR-1 stimulates expression of a series of atherogenic genes including JE/MCP, IL-1b, tissue factor (TF), plasminogen activator inhibitor (PAI-1), VCAM-1 and ICAM-1. EGR-1 activity seems not to affect cholesterol and triacylglycerol levels [52]. It is enriched in fibrous caps of atherosclerotic plaques [54]. The EGR-1-dependent gene products TF and PAI-1 are prothrombotic and associated with fibrin deposition in these atheromatous areas thus enhancing plaque instability and rupture [15].

OxLDL stimulates EGR-1 expression in RAW macrophages in a dose-dependent manner. OxLDL-dependent EGR-1 expression is mediated by the MEK-ERK1/2 MAP kinase pathway. RAW cells pretreated with the MEK-1 inhibitorPD98059 do not express EGR-1 if challenged with oxidized lipoprotein [52]. From our microarray data, it can be concluded that PGPC and POVPC contribute to the inflammatory procoagulant activity of oxLDL as pertinent to EGR-1. However, it remains open whether POVPC and PGPC utilize the same signaling pathways to trigger these effects.

### PGPC, atherogenesis and inflammation

PGPC upregulated expression of the proatherogenic and inflammatory genes Cd9, Src kinase, c-Jun and JunB. POVPC did not affect these genes in our study. They are related to scavenger receptor-mediated uptake of oxLDL in macrophages and its intracellular signaling, since they act cooperatively with CD36. CD36 is the major receptor for oxLDL in these cells. Scavenger receptor A may also be involved in this process although to a lesser extend [55]. CD36-mediated signaling in response to oxPLs may follow two routes. After lipoprotein internalization, its oxidized lipid components can directly bind to nuclear receptors thereby triggering gene expression [46]. Alternatively, CD36 activates a signalling cascade involving Src kinases, the MAP kinases c-Jun N-terminal kinase-1 and −2, and finally the AP-1 components c-Jun and JunB [56], [57] (for activities of AP-1 see inflammatory and apoptotic response to PGPC). CD36 signalling in response to oxLDL is directly modulated by CD9 which binds to the cytoplasmic side of the receptor [58]. It has to be noted that the effects described above are cell-specific. For instance, the response of peritoneal macrophages to CD36 are mediated by the Src kinase lyn and JNK, whereas fyn and the MAP kinase p38 are involved in endothelial cells [56].

CD36-mediated oxLDL uptake by macrophages is not regulated and leads to accumulation of toxic lipoprotein material inside the cells. This process induces the formation of foam cells which is a hallmark of atherosclerosis. In the literature it is emphasized that modification of positively charged lysines of apoB by lipid oxidation products (aldehydes) is responsible for the switch from the “beneficial“ apoB receptor to the ”detrimental“ scavenger receptor. A similar function has also been attributed to PGPC. This oxPL directly confers net negative charges to the LDL surface and thus makes it prone to CD36 binding and its consecutive intracellular signaling [6]. On top of that, PGPC enhances oxLDL activity by upregulating important genes that propagate its signals.

### PGPC and regulation of cell cycle

PGPC downregulated the expression of the cell cycle-dependent proteins Cyclin B1, Cyclin F, Cyclin A2, Cdc5, Cdca7, Cdc6 and PLK1. It has been reported that oxLDL elicits similar effects in vascular smooth muscle cells and fibroblasts [59]. Thus, PGPC is likely to contribute to the effects of the modified lipoprotein on the cell cycle, too. PLK1 seems to play a particular role in this respect. This kinase is a member of the protein family PLK1–4, that promotes entry of the cell cycle to mitosis. It induces degradation of Wee1 kinase which phosphorylates and thereby inhibits the Cdc2/Cyclin B1 complex (maturation factor). As a consequence, the cell enters cell cycle arrest and eventually becomes subject to apoptosis which represents the endpoint of oxPL toxicity in macrophages [60], [61].

### Conclusion

OxLDL is an atherogenic particle that contains a large variety of biologically active lipids and modified apolipoprotein epitopes that are responsible for its activity. This study made an attempt to identify the contributions of two major oxidized phospholipids, namely PGPC and POVPC that localize to the surface of the particle. We determined the effects of the chemically defined compounds on gene expression in cultured macrophages and found that they largely contribute to oxLDL activities that are responsible for its atherogenicity (see Figure 4). OxLDL contains in addition to PGPC and POVPC a large variety of other oxidized phospholipids with potential and perhaps different bioactivity. Many of these compounds still await the development of (bio)chemical methods to prepare sufficient amounts for biomedical research.

## Supporting Information

File S1 **Effects of PGPC and POVPC on gene expression in RAW 264.7 macrophages.** Transcription analysis was performed using a genome-wide microarray containing 27648 oligonuceotide probes for murine genes (http://www.genome.tugraz.at/adipocyte/Microarray.html). Cells were incubated with oxPLs for the indicated times, cDNA was prepared and microarray analysis was performed as described in “Materials and Methods”. Shown are log_2_-fold changes of gene expression of all affected genes for oxPL-treated cells compared to oxPL-free controls.
(DOCX)
