# Supplementary material for: Effects of Oxidized Phospholipids on Gene Expression in RAW 264.7 Macrophages: A Microarray Study
Source: PLoS One. 2014 Oct 21;9(10):e110486. doi: 10.1371/journal.pone.0110486 (PMC4204898; doi:10.1371/journal.pone.0110486)
Supplement: File S1 — Effects of PGPC and POVPC on gene expression in RAW 264.7 macrophages. Transcription analysis was performed using a genome-wide microarray containing 27648 oligonuceotide probes for murine genes (http://www.genome.tugraz.at/adipocyte/Microarray.html). Cells were incubated with oxPLs for the indicated times, cDNA was prepared and microarray analysis was performed as described in “Materials and Methods”. Shown are log2-fold changes of gene expression of all affected genes for oxPL-treated cells compared to oxPL-free controls. (DOCX) [file pone.0110486.s001.docx]

|  | **log_2_-fold changes of gene expression** | | | | | |
| --- | --- | --- | --- | --- | --- | --- |
|  | **PGPC** | | | **POVPC** | | |
| **Gene symbol** | **1 h** | **2 h** | **4 h** | **1 h** | **2 h** | **4 h** |
| Jun | **2,20** | **2,43** | **1,32** | **0,66** |  |  |
| Reps2 | **1,56** | **2,72** | **1,66** |  |  |  |
| Klf6 | **1,74** | **2,63** | **1,85** | **0,68** | **0,84** |  |
| Rhob | **2,13** | **1,79** | **2,72** |  |  |  |
| Hmgb3 | **2,06** |  | **1,36** | **0,70** |  |  |
| Txnip | **1,48** |  |  |  |  |  |
| Cry2 | **0,88** | **1,34** |  |  |  |  |
| Atf4 | **0,88** | **1,03** | **0,79** |  |  |  |
| Helb | **1,87** | **2,03** | **0,63** |  |  |  |
| Cd36 | **0,82** |  |  |  |  |  |
| Tsc22d3 | **0,73** | **0,91** | **1,14** |  |  |  |
| Egr1 | **2,18** | **2,39** | **0,78** |  | **0,59** |  |
| Myd116 | **0,88** |  | **0,82** |  |  |  |
| 2610101N10Rik | **0,65** |  | **0,77** |  |  |  |
| Pnrc1 | **0,62** | **0,70** |  |  |  |  |
| Klf2 | **1,07** | **1,06** | **1,37** |  |  |  |
| Atf3 | **0,92** |  | **2,95** |  | **0,96** | **0,72** |
| Oaz2 | **1,08** | **1,79** | **1,51** |  |  |  |
| Slc17a7 | **0,58** |  | **0,74** |  |  |  |
| Scfd2 | **0,83** | **1,01** | **0,73** |  |  |  |
| Rgs2 | **0,62** |  | **0,88** |  | **0,70** |  |
| Mrpl55 | **0,77** | **0,83** | **1,04** |  |  |  |
| Nploc4 | **0,67** | **0,86** | **0,99** |  |  |  |
| Clk1 | **0,71** | **0,78** |  |  |  |  |
| Mbp | **0,66** |  | **1,16** |  |  |  |
| 2900083I11Rik | **0,58** |  |  |  |  |  |
| Scmh1 | **0,63** | **0,71** |  |  |  |  |
| Tars | **0,69** |  | **1,30** |  |  |  |
| Slco3a1 | **0,76** |  | **0,99** |  |  |  |
| Ifrd1 | **0,67** | **1,00** | **1,55** |  |  |  |
| Ubc\|Ubb | **0,73** |  |  |  |  |  |
| D430041D05Rik | **0,67** | **1,21** |  |  |  |  |
| Sp3 | **0,63** |  |  |  |  |  |
| Cxcl1 | **0,74** | **0,99** | **1,86** |  |  |  |
| Prkcb1 | **0,60** |  |  |  |  |  |
| Emp1 |  | **1,33** | **2,11** |  | **0,58** |  |
| Cttnbp2nl |  | **0,90** | **0,94** |  |  |  |
| Rasgef1b |  | **1,10** |  |  |  |  |
| Mdm2 |  | **0,81** | **0,59** |  |  |  |
| Zfp655 |  | **0,72** |  |  |  |  |
| Tmem49 |  | **0,65** | **0,83** |  |  |  |
| Gp49a\|Lilrb4 |  | **0,67** | **0,96** |  |  |  |
| Mdfic |  | **0,59** | **0,64** |  |  |  |
| Cotl1 |  | **1,12** | **2,13** |  |  |  |
| Pcf11 |  | **0,59** |  |  |  |  |

|  | **PGPC** | | | **POVPC** | | |
| --- | --- | --- | --- | --- | --- | --- |
| **Gene symbol** | **1 h** | **2 h** | **4 h** | **1 h** | **2 h** | **4 h** |
| Bet1 |  | **0,77** |  |  |  |  |
| Trib3 |  | **1,15** | **0,59** |  |  |  |
| Serpina3g\|Serpina3h\|Serpina3f\|Serpina3n |  | **0,74** |  |  |  |  |
| Wdr5 |  | **0,61** |  |  |  |  |
| Wsb1 |  | **0,65** |  |  |  |  |
| Abl2 |  | **0,61** | **0,76** |  |  |  |
| Slc2a4 |  | **0,59** | **0,60** |  |  |  |
| Klf7 |  | **1,01** | **0,87** |  |  |  |
| Gja1 |  | **0,83** | **0,66** |  |  |  |
| Smg7 |  | **0,61** |  |  |  |  |
| Zfand5 |  | **0,72** | **0,71** |  |  |  |
| Egr4\|Egr3\|Egr2 |  | **0,68** |  |  |  |  |
| Tank |  | **0,66** | **0,89** |  |  |  |
| Fbxw17 |  | **0,94** |  |  |  |  |
| RP24-320O9.1\|1700029I01Rik\|OTTMUSG00000010657 |  | **0,88** |  |  |  |  |
| Rcan1 |  | **0,77** |  |  |  |  |
| Klf4 |  | **0,87** | **1,47** |  |  |  |
| Trim6 |  | **0,74** |  |  |  |  |
| Junb |  | **0,63** | **0,70** |  |  |  |
| Wdr26 |  | **0,75** | **0,90** |  |  |  |
| Sat1 |  | **0,92** | **1,96** |  |  |  |
| Tnf |  | **0,70** | **1,08** |  |  |  |
| Zdhhc21 |  | **0,83** |  |  |  |  |
| Klhl26 |  | **0,79** |  |  |  |  |
| Cdkn2aip |  | **-0,96** |  |  |  |  |
| Gprc5a |  |  | **1,37** |  |  |  |
| Pdgfb |  |  | **2,01** |  |  | **0,71** |
| Plaur |  |  | **1,39** |  |  |  |
| BC004044 |  |  | **1,42** |  |  | **0,69** |
| Cd9 |  |  | **1,02** |  |  |  |
| Ywhah |  |  | **1,09** |  |  |  |
| Tnfrsf12a |  |  | **1,07** |  |  |  |
| Plekho2 |  |  | **1,13** |  |  |  |
| Pctk3 |  |  | **1,01** |  |  |  |
| Anxa2 |  |  | **0,93** |  |  |  |
| D930005D10Rik |  |  | **0,92** |  |  |  |
| Slc20a1 |  |  | **1,30** |  |  |  |
| ENSMUSG00000072907\|Oog1\| OTTMUSG00000010009 |  |  | **0,86** |  |  |  |
| Mesp2 |  |  | **0,84** |  |  |  |
| 1200015F23Rik |  |  | **1,03** |  |  |  |
| Cenpb |  |  | **0,89** |  |  |  |
| Micall1 |  |  | **1,14** |  |  |  |
| 6430527G18Rik |  |  | **0,90** |  |  |  |
| Iqgap1 |  |  | **0,82** |  |  |  |
| Cdc42ep2 |  |  | **0,85** |  |  |  |
| Slc2a1 |  |  | **0,83** |  |  |  |

|  | **PGPC** | | | **POVPC** | | |
| --- | --- | --- | --- | --- | --- | --- |
| **Gene symbol** | **1 h** | **2 h** | **4 h** | **1 h** | **2 h** | **4 h** |
| Myadm |  |  | **0,96** |  |  |  |
| Ier3 |  |  | **1,15** |  |  |  |
| Bmpr1a |  |  | **0,78** |  |  |  |
| Nfkbia |  |  | **1,12** |  |  |  |
| Cep170 |  |  | **0,72** |  |  |  |
| Ets2 |  |  | **1,01** |  |  |  |
| Ctdspl |  |  | **0,82** |  |  |  |
| Slc1a2 |  |  | **1,07** |  |  |  |
| Clic4 |  |  | **0,83** |  |  |  |
| Rtn4 |  |  | **0,70** |  |  |  |
| Blcap |  |  | **0,70** |  |  |  |
| Fgd6 |  |  | **0,73** |  |  |  |
| 9130404D14Rik |  |  | **0,84** |  |  |  |
| Flna |  |  | **0,74** |  |  |  |
| Slc35a4 |  |  | **0,71** |  |  |  |
| Cdc42ep4 |  |  | **0,66** |  |  |  |
| 2900011O08Rik |  |  | **0,78** |  |  |  |
| Tnfsf12 |  |  | **0,66** |  |  |  |
| Plec1 |  |  | **0,87** |  |  |  |
| Pmp22 |  |  | **0,69** |  |  |  |
| Anxa7 |  |  | **0,67** |  |  |  |
| Smad1 |  |  | **0,69** |  |  |  |
| Ptk2b |  |  | **0,67** |  |  |  |
| Ldlr |  |  | **0,71** |  |  |  |
| Cd44 |  |  | **0,80** |  |  |  |
| Phldb1 |  |  | **0,65** |  |  |  |
| Adrb2 |  |  | **0,96** |  |  |  |
| Psmd8 |  |  | **0,66** |  |  |  |
| Stab1 |  |  | **0,83** |  |  |  |
| Klf3 |  |  | **0,78** |  |  |  |
| Trpv2 |  |  | **0,61** |  |  |  |
| Cd14 |  |  | **0,82** |  |  |  |
| Pcyt1a |  |  | **0,65** |  |  |  |
| Ehd1 |  |  | **0,71** |  |  |  |
| Cpeb4 |  |  | **0,95** |  |  |  |
| 1110005A03Rik |  |  | **0,69** |  |  |  |
| Adamtsl5 |  |  | **0,72** |  |  |  |
| Slc23a2 |  |  | **0,65** |  |  |  |
| Pfkp |  |  | **0,58** |  |  |  |
| Gadd45b |  |  | **0,67** |  |  |  |
| S100a10 |  |  | **0,65** |  |  |  |
| Klf9 |  |  | **0,86** |  |  |  |
| Rhbdf1 |  |  | **0,61** |  |  |  |
| Src |  |  | **0,88** |  |  |  |
| Cd97 |  |  | **0,89** |  |  |  |
| Prkcc |  |  | **0,59** |  |  |  |

|  | **PGPC** | | | **POVPC** | | |
| --- | --- | --- | --- | --- | --- | --- |
| **Gene symbol** | **1 h** | **2 h** | **4 h** | **1 h** | **2 h** | **4 h** |
| Pik3c3 |  |  | **0,58** |  |  |  |
| Pdcd10 |  |  | **0,76** |  |  |  |
| Ndrg1 |  |  | **0,87** |  |  |  |
| Srgn |  |  | **0,69** |  |  |  |
| Apba1 |  |  | **0,68** |  |  |  |
| Vim |  |  | **0,60** |  |  |  |
| Mpeg1 |  |  | **0,64** |  |  |  |
| Phf8 |  |  | **0,83** |  |  |  |
| Ncan |  |  | **0,61** |  |  |  |
| Tgfbr1 |  |  | **0,99** |  |  |  |
| Sdhd |  |  | **0,63** |  |  |  |
| Sostdc1 |  |  | **0,70** |  |  |  |
| Rhoc |  |  | **0,98** |  |  |  |
| B3gnt2 |  |  | **0,66** |  |  |  |
| Ahnak2 |  |  | **0,59** |  |  |  |
| Emp3 |  |  | **0,66** |  |  |  |
| Nip7 |  |  | **0,75** |  |  |  |
| Lcp2 |  |  | **0,59** |  |  |  |
| Lims2 |  |  | **0,64** |  |  |  |
| Txn2 |  |  | **0,62** |  |  |  |
| Anxa1 |  |  | **0,71** |  |  |  |
| Dhrs3 |  |  | **0,62** |  |  |  |
| Maged1 |  |  | **0,88** |  |  |  |
| Stx3 |  |  | **0,63** |  |  |  |
| Rapgef2 |  |  | **0,61** |  |  |  |
| Scn2b |  |  | **0,58** |  |  |  |
| Fem1b |  |  | **0,62** |  |  |  |
| Myo1b |  |  | **0,68** |  |  |  |
| AK122525 |  |  | **0,65** |  |  |  |
| Sqstm1 |  |  | **0,80** |  |  |  |
| Ralgds |  |  | **0,59** |  |  |  |
| Kcnk6 |  |  | **0,68** |  |  |  |
| Rap1b |  |  | **0,72** |  |  |  |
| Btg1 |  |  | **0,58** |  |  |  |
| Hmga2 |  |  | **0,89** |  |  |  |
| Coq10b |  |  | **0,61** |  |  |  |
| Pfpl |  |  | **0,59** |  |  |  |
| Tcf19 |  |  | **-1,03** |  |  |  |
| Cdca5 |  |  | **-0,80** |  |  |  |
| Rpa2 |  |  | **-0,80** |  |  |  |
| Ccnb1 |  |  | **-0,74** |  |  |  |
| Hist3h2a |  |  | **-0,83** |  |  |  |
| Uhrf1 |  |  | **-0,77** |  |  |  |
| Rad51 |  |  | **-0,67** |  |  |  |
| Top2a |  |  | **-0,74** |  |  |  |
| Cubn |  |  | **-0,64** |  |  |  |

|  | **PGPC** | | | **POVPC** | | |
| --- | --- | --- | --- | --- | --- | --- |
| **Gene symbol** | **1 h** | **2 h** | **4 h** | **1 h** | **2 h** | **4 h** |
| Ccne1 |  |  | **-0,98** |  |  |  |
| Cdca7 |  |  | **-0,65** |  |  |  |
| Hist1h2ao\|Hist1h2ag\|Hist1h2ad\|Hist1h2ai\| Hist1h2ac\|Hist1h2af\|Hist1h2ah |  |  | **-0,72** |  |  |  |
| Fen1 |  |  | **-0,65** |  |  |  |
| Dtl |  |  | **-0,91** |  |  |  |
| Cdc6 |  |  | **-0,60** |  |  |  |
| Usp1 |  |  | **-0,75** |  |  |  |
| Asf1a |  |  | **-0,68** |  |  |  |
| Cbx5 |  |  | **-0,63** |  |  |  |
| Abca1 |  |  | **-1,10** |  |  | **-0,72** |
| Ccna2 |  |  | **-0,71** |  |  |  |
| Hist1h4m\|Hist1h4a\|Hist1h4h\|Hist1h4b\| Hist1h4i\|Hist1h4f\|Hist1h4k\|Hist1h4j\|Hist1h4d |  |  | **-0,77** |  |  |  |
| Cep55 |  |  | **-0,64** |  |  |  |
| Chaf1b |  |  | **-0,66** |  |  |  |
| Ttk |  |  | **-0,58** |  |  |  |
| Plk1 |  |  | **-0,62** |  |  |  |
| Aspm |  |  | **-0,62** |  |  |  |
| Nlrp14 |  |  | **-0,59** |  |  |  |
| Aurka |  |  | **-0,68** |  |  |  |
| Sema6c |  |  | **-0,77** |  |  |  |
| Chst10 |  |  | **-0,63** |  |  |  |
| Exosc9 |  |  | **-0,83** |  |  |  |
| D10Wsu52e |  |  | **-0,60** |  |  |  |
| Sfrs3 |  |  | **-0,60** |  |  |  |
| Aurkc |  |  | **-0,77** |  |  |  |
| Ncaph |  |  | **-0,68** |  |  |  |
| Ccnf |  |  | **-0,69** |  |  |  |
| Gclc |  |  | **-0,58** |  |  |  |
| Ppil1 |  |  | **-0,59** |  |  |  |
| Mcm6 |  |  | **-0,67** |  |  |  |
| Prei4 |  |  | **-0,60** |  |  |  |
| Pscdbp |  |  | **-0,69** |  |  |  |
| 2810417H13Rik |  |  | **-0,67** |  |  |  |
| Arnt |  |  | **-0,70** |  |  |  |
| Zfp518b |  |  | **-0,65** |  |  |  |
| Deaf1 |  |  | **-0,62** |  |  |  |
| Hist1h1c\|Hist1h1d\|Hist1h1e\|Hist1h1b |  |  | **-0,76** |  |  |  |
| Klc1 |  |  | **-0,67** |  |  |  |
| Mt2 |  |  | **-0,67** |  |  |  |
| B4galt5 |  |  |  |  | **-0,60** |  |
| Lpl |  |  |  |  |  | **0,62** |
